# Identification of a novel stripe rust resistance gene from the European winter wheat cultivar ‘Acienda’: A step towards rust proofing wheat cultivation

**DOI:** 10.1371/journal.pone.0264027

**Published:** 2022-02-16

**Authors:** Gomti Grover, Achla Sharma, Ian Mackay, Puja Srivastava, Satinder Kaur, Jaspal Kaur, Amanda Burridge, Sacha Przewieslik Allen, Alison R. Bentley, Parveen Chhuneja, N. S. Bains

**Affiliations:** 1 Punjab Agricultural University, Ludhiana, India; 2 IMPlant Consultancy Ltd., Chelmsford, United Kingdom; 3 Life Sciences, University of Bristol, Bristol, United Kingdom; 4 The John Bingham Laboratory, NIAB, Cambridge, United Kingdom; Julius Kuhn-Institut, GERMANY

## Abstract

All stage resistance to stripe rust races prevalent in India was investigated in the European winter wheat cultivar ‘Acienda’. In order to dissect the genetic basis of the resistance, a backcross population was developed between ‘Acienda’ and the stripe rust susceptible Indian spring wheat cultivar ‘HD 2967’. Inheritance studies revealed segregation for a dominant resistant gene. High density SNP genotyping was used to map stripe rust resistance and marker regression analysis located stripe rust resistance to the distal end of wheat chromosome 1A. Interval mapping located this region between the SNP markers AX-95162217 and AX-94540853, at a LOD score of 15.83 with a phenotypic contribution of 60%. This major stripe rust resistance locus from ‘Acienda’ has been temporarily designated as *Yraci*. A candidate gene search in the 2.76 Mb region carrying *Yraci* on chromosome 1A identified 18 NBS-LRR genes based on wheat RefSeqv1.0 annotations. Our results indicate that as there is no major gene reported in the *Yraci* chromosome region, it is likely to be a novel stripe rust resistance locus and offers potential for deployment, using the identified markers, to confer all stage stripe rust resistance.

## Introduction

Global wheat demand is projected to rise at an annual rate of 1.6% to 2050 on account of increasing population and income levels [1]. Wheat is vulnerable to a number of biotic and abiotic stresses depending on the agro-climatic zones. Stripe rust caused by *Puccinia striiformis* f. sp. *tritici*, (*Pst*) is an economically important foliar disease prevalent in all major wheat growing regions and considered the most destructive disease of wheat throughout the world [2]. It poses a significant threat to wheat productivity, leading to significant yield losses.

Historically stripe rust has caused epidemics in cool and moist regions, but it is now more frequently reported in warmer and drier areas suggesting an expansion in its range of adaptation [3–6]. Its increasing importance as a destructive wheat pathogen is also likely due to continuous evolution of new virulences [7]. A recent study estimated that 88% of the world’s wheat production is now prone to stripe rust infection, resulting in global losses of at least 5 million tonnes annually [6].

Stripe rust can be controlled by the application of fungicides but this increases the costs of wheat production and can be ineffective if not applied at correct time [8]. The widespread use of fungicides also leads to negative environmental effects due to chemical residues. Therefore, there is a clear need to focus on deploying and updating genetic resistance in wheat varieties against evolving stripe rust races. Characterisation of genetic resistance for stripe rust was first described in the wheat cultivar Rivet [9] and remains an active process in global wheat research [10, 11]. Currently, more than 70 stripe rust resistance genes, designated through *Yr1* to *Yr76*, have been catalogued in different hexaploid bread, durum wheat, and wild species backgrounds [11]. However, progress in the discovery and introgression of novel genes and alleles is typically hampered by the availability of identified donor stocks with proven resistances, the long time frames necessary for identifying even monogenic resistances, and the disconnect between gene identification and mobilization in breeding. Biotechnological approaches help in elucidating the molecular mechanism of resistance. With the advent of DNA-based molecular markers, plant breeding with the aid of marker assisted selection (MAS) has given new dimensions to crop improvement. Characterization of genes and identification of linked molecular markers are powerful tools in deployment of desirable traits into elite background.

A single dominant stripe rust resistance source in the European bread wheat cultivar ‘Acienda’ was reported in our previous report [12]. In the present study, we report molecular mapping of this all-stage stripe rust resistance gene using high throughput SNP genotyping in backcross populations. This new gene offers an additional source of stripe rust resistance supporting the breeding of future disease resistant wheat cultivars.

## Results

### Phenotypic analysis indicates monogenic control of stripe rust resistance

Both BC_1_F_2_ populations conformed to a segregation ratio of 3 resistant (R) to 1 susceptible(S) (3R:1S), indicating a single dominant stripe rust resistance locusfrom Acienda (Table 1). Progeny test in BC_1_F_3_ showed segregation in a 1:2:1 ratio again confirming dominant monogenic inheritance (Table 1).

**Table 1:** Segregation and chi-square analysis of resistance in BC_1_F_2_ plants and BC_1_F_3_ progeny indicates segregation of single dominant locus.

Population @	No. of BC_1_F_2_ plants			No. of BC_1_F_3_ progeny^#^			
	Resistant	Susceptible	χ^2^ (3:1)	HR	Seg.	HS	χ^2^ (1:2:1)
P1	68	18	0.76	22	46	18	0.79
P2	88	18	3.64	28	56	18	2.94

^@^derived from two BC_1_F_1_ plants

^#^HR: homozygous resistant; Seg: segregating; HS: homozygous susceptible.

### Molecular mapping confirms presence of a major resistance locus on chromosome 1A

**Fig 1:**
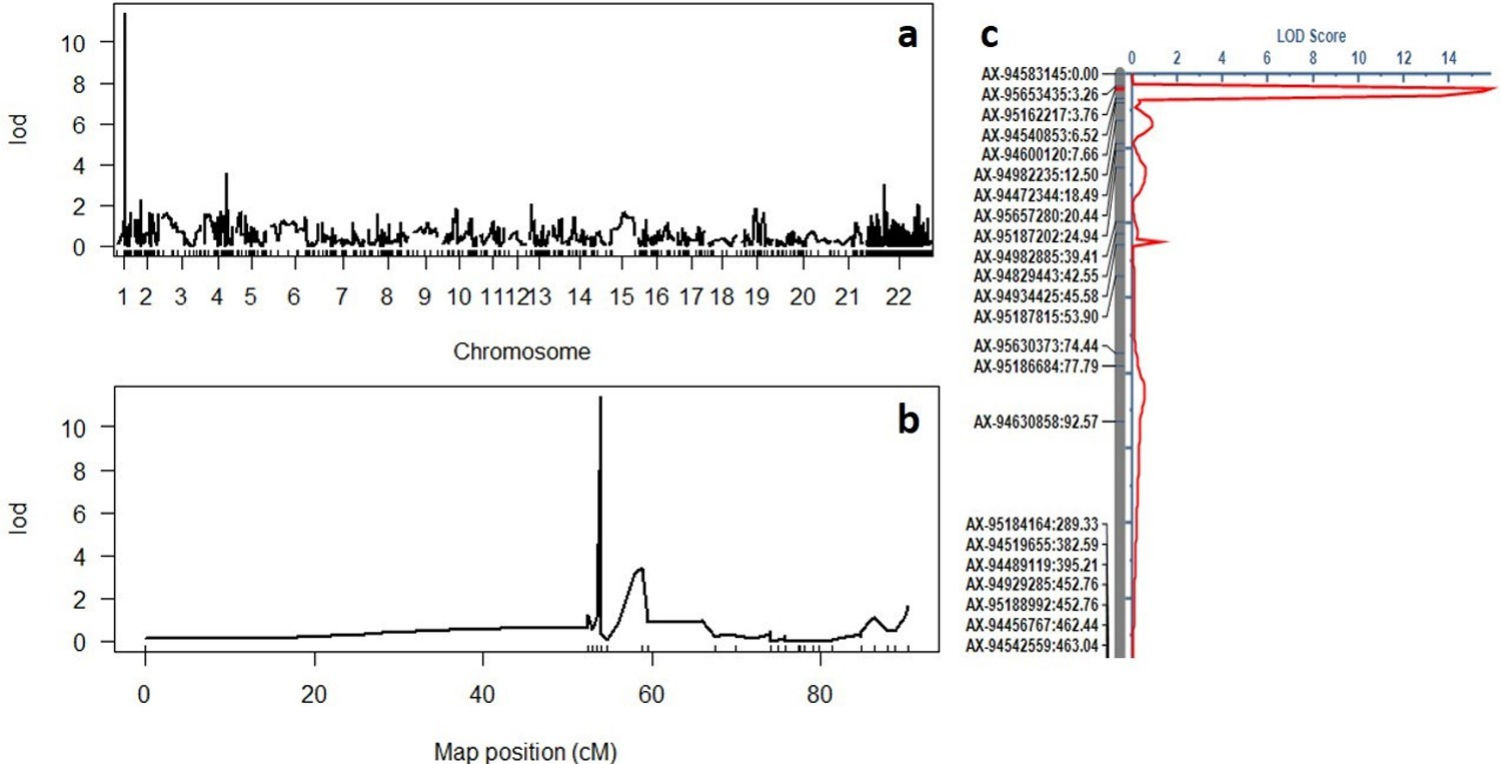
(a) Genome wide marker trait associations for stripe rust resistance in Acienda/HD2967 BC_1_F_3_ population generated in R/qtl showing a single large peak on chromosome 1A. (b) LOD scores for chromosome 1A depicting mapping of *Yraci* (c) Mapping of stripe rust resistance in Acienda/HD2967 population on wheat chromosome 1A using ICI Mapping software. Physical locations of the markers are indicated in Mb.

Out of 35,143 SNP markers of wheat breeders’ array, 2,359 polymorphic SNP markers were used for mapping stripe rust resistance at BC_1_F_3_. Detail of filtering of 35K SNP markers were described in material method section. These 2359 markers were distributed across all 21 wheat chromosomes as summarised in S1 Table in S1 File. Marker regression analysis was performed using the Haley-Knott method as implemented in R/qtl. This detected one prominent peak on chromosome 1A flanked by SNP marker AX-95162217 at a LOD score of 11.4 (Fig 1A). Two minor peaks were also observed on chromosome 2A but these were below the LOD threshold (3.5 and 3.0, respectively) and were not considered further. Some markers which could not be assigned to any of the linkage groups remained unmapped.

Single marker analysis (SMA) was also performed using the ICI mapping which confirmed the mapping of stripe rust resistance in the interval flanked by SNP markers AX-94540853 and AX-95162217 at a LOD score of 16.99. Interval mapping with ICIM detected major QTL flanked by SNP markers AX-95162217 and AX-94540853 with a LOD score of 15.83 explaining 60% of the phenotypic variation (Fig 1C). Based on the existing consensus genetic map (Allen et al. 2017), AX-95162217 and AX-94540853 were mapped in the bin at 54.04cM on chromosome 1A. This new stripe rust resistance locus on 1A is designated as *Yraci*.

Physical locations of the SNP markers linked to *Yraci* were obtained by BLAST search against the IWGSC Ref Seq v1 genome assembly (IWGSC, 2018). A physical interval of 2.76 Mb between markers AX-95162217 (3.76Mb) and AX-94540853 (6.52Mb) defined the position of *Yraci*. Candidate genes in this 2.76 Mb region harbouring *Yraci*, were identified from IWGSC Ref Seq v1.0 annotation which revealed a total of 18 NBS-LRR genes in this region (S2 Table in S1 File).

## Discussion

In this study we report the phenotypic and genetic characterisation of a stripe rust resistance locus *Yraci* from the European winter wheat cultivar ‘Acienda’. Analysis of segregation patterns of resistance in backcross populations developed from ‘Acienda’ and the Indian spring wheat ‘HD 2967’ support monogenic inheritance of the locus, confirming the previous findings in [12]. Genotyping the backcross population with the high-density 35K Affymetrix Wheat Breeders SNP array produced 2,359 informative markers for mapping. The low number of polymorphic markers identified for the population most likely reflected the backcross derived nature of the material used in the study. The backcross with ‘HD2967’ was primarily performed for mobilization of resistance into the elite and adapted agronomic background. Moreover, the synchronization of the phenological stages for uniform disease evaluation could also be achieved to ensure uniform disease evaluation.

Genetic characterization identified a single locus on chromosome 1A explaining 60% of the phenotypic variation in resistance. This was assigned as the stripe rust resistance locus Y*raci* located in a 2.76 Mb interval between SNP markers AX-95162217 and AX-94540853 on the distal end of chromosome 1A. No major stripe rust resistance gene has been previously reported in this region although previous studies have detected minor quantitative trait loci (QTL). Based on reports in literature, ‘Janz’, an Australian cultivar susceptible to *Pst* isolates at the seedling stage showed an adult plant stripe rust resistance response [13] which was mapped to a minor QTL from ‘Janz’ on chromosome 1A, explaining 6–7% of the phenotypic variation and designated as *QYr*.*sun-1A*. A minor QTL on 1A (*QYrid*.*ui-1A*) responsible for high-temperature adult-plant resistance to stripe rust has also been reported from the resistant source ‘IDO444’ [14]. A further QTL (*QYr*.*tam-1AS*) derived from ‘TAM 111’, a hard red winter wheat cultivar resistant to *Pst* races in the southern-central USA was reported to be located on 1AS chromosome of wheat, explaining 17% phenotypic variation [15]. These previous putative stripe rust resistance loci indicate that minor QTLs for adult plant resistance reside on chromosome 1A chromosome. The mapping of a major effect all stage resistance locus on chromosome 1A in this study suggests that *Yraci* is a novel resistance source that has not yet been exploited for spring wheat improvement.

The available Ref Seq v1.0 wheat genome annotation (IWGSC, 2018) allowed the identification of 18 candidate NBS-LRR family genes in the mapped interval. Disease resistance genes are typically found in clusters and use of physical distance (rather than genetic distance) permitted the narrowing of the region. Further work is required to develop markers for characterising and tracking these specific gene for use in resistance breeding. This is crucial as accelerated evolution of stripe rust pathotypes have been reported in India. The appearance of race 78S84 which has virulence for *Yr27* [16] broke down the resistance of ‘PBW343’, a cultivar widespread in India for over a decade (1995–2007). In addition, two newly evolved pathotypes (110S119 first reported in 2014 from Ropar, Punjab, India; 238S119 first reported in 2014 from Himachal Pradesh, India) have led to the breakdown of resistance in breeding pipelines as well in the cultivars released after ‘PBW343’ [17, 18].

This catalysed wheat breeders and wheat pathologists to mobilise known gene resistance into agronomically acceptable backgrounds and also to identify novel sources of resistance to be used in future. Currently, resistance breeding depends on a relatively small number of resistance genes (*Yr5*, *Yr10*, *Yr15*, *Lr37*/*Yr17* and *Lr76*/*Yr70)* which are being used in various, but often limited. combinations. This limitation presents an urgent need to diversify the available resistant sources. We demonstrate that *Yraci* is a new locus offering additional support to wheat breeders for developing durable stripe rust resistance.

The European winter wheat variety Acienda was registered in France in 2004 and postulated to have *Lr13* leaf rust resistant gene [19]. Leaf rust is the major foliar disease of wheat in France, and by 2007 the majority of French cultivars had two (*Lr13*, *Lr37*) or three (*Lr10*, *Lr13*, *Lr37*) gene combinations. In India, Acienda showed strong resistance to all prevalent stripe rust races and pathotypes in field screening experiments. Further, it did not show presence of other effective stripe rust resistance genes currently in use in India when screened with available linked molecular markers [12]. Via backcross introgression into an agronomically elite Indian spring wheat background we mapped the novel *Yraci* locus to chromosome 1A and created advanced germplasm carrying the locus for use in breeding. This represents a new source of stripe rust resistance for the region and the germplasm developed is an important resource for future wheat breeding and improvement.

## Materials and methods

### Plant material

The European winter wheat cultivar ‘Acienda’ was identified as having all stage stripe rust resistance in field trials at Punjab Agricultural University (PAU), Ludhiana, India (30° 54’ N latitude, 75° 48’ E longitude, and 247m above m s l) over five seasons (2011–2016) [12]. The resistant donor parent ‘Acienda’ was selected from the 376-line European winter wheat Triticeae Genome association mapping panel [20]. ‘Acienda’(Tremie/Sideral), is a cultivar with winter growth habit that was released in France in 2004 and was widely grown [19, 21]. In order to map the resistance, ‘Acienda’ was crossed with stripe rust susceptible spring wheat cultivar, ‘HD 2967’ and the F_1_ plants backcrossed to ‘HD 2967’. Wheat variety ‘HD2967’ is currently the most widely grown cultivar (based on seed production) in India [18]. The BC_1_F_1_ plants were screened for stripe rust under artificial epiphytotic conditions in the field and resistant BC_1_F_1_ plants were individually harvested. Further the individual BC_1_F_2_ seeds were space planted in the field and screened for stripe rust resistance. Two BC_1_F_2_ populations (P1 and P2) with 86 and 106 plants each, respectively (derived from different BC_1_F_1_ plants) were used. The BC_1_F_2_ plants from each population were harvested individually and planted as BC_1_F_3_ progeny rows. The backcross derived material was screened for stripe rust resistance. A schematic representation of population development is given in S1 Fig.

### Field inoculation & resistance phenotyping

Plant material was inoculated with the mixture of major stripe rust pathotypes *Pst* 78S84, *Pst* 46S119, 110S119 and 238S119 [17]. Primary inoculations and disease pressure were built up as per the standard protocol [12]. Infector rows of the susceptible cultivars ‘PBW 343’ and ‘HD 2967’ were planted at the borders of each population to ensure disease pressure.

The severity of stripe rust infection was recorded on individual BC_1_F_1_, BC_1_F_2_ plants and BC_1_F_3_plantsusing a modified Cobb’s scale [22]. Disease progress was recorded at three time points with the first observation two weeks after inoculation and second at three weeks post-inoculation. Final disease severity was recorded when susceptible parents recorded the highest disease severity (80S or above). For genetic analysis, the BC_1_F_2_ plants were categorized in two classes based on individual disease reaction score. Plants with disease severity score of Ts to 5S were considered as resistant based upon the score of resistant donor ‘Acienda’ and plants with a score of 20S or were considered as susceptible. A Chi-squared test was used to test for goodness of fit with Mendelian expectations.

### Genotyping

Bulked DNA of 86 BC_1_F_3_ progenies from P1 were genotyped along with the parents. Genomic DNA from the leaves of individual plants of two weeks old seedlings of BC_1_F_3_ progenies were extracted using the standard CTAB (Cetyl trimethyl ammonium bromide) procedure. Equal quantity of DNA of individual plants of each BC_1_F_3_ progeny were bulked to represent BC_1_F_2_ genotype. DNA quantity was determined using a spectrophotometer and rechecked on 0.8% agarose gels. Seven DNA samples did not pass the quality control parameters so in total 79 progenies from the population were genotyped. Approximately 200ng DNA from each sample was used for genotyping using the Axiom® Wheat Breeders’ Array [23] which contains 35,143 SNPs using the Affymetrix GeneTitan® system (Affymetrix Axiom® 2.0 Assay for 384 samples P/N 703154 Rev. 2). The allele calling was performed using Affymetrix Analysis Suite (version 1.1.0.616) under polyploid parameters. A Dish QC of 0.8 was used with a 90% call rate cut-off for sample QC prior to allele calling.

The resulting SNP data was filtered by removing 5,667 SNP markers with more than 12% missing data and 16,321 monomorphic SNP markers. In addition, 836 SNP markers giving heterozygous calls in all samples and 9,960 markers with minor allele frequency less than 9.5 were also removed. A final set of 2,359 polymorphic SNP markers were used for further analysis.

### Statistical analysis

Chi-squared analyses were performed to determine the goodness of fit of observed segregations for stripe rust to establish the number and nature of gene(s) governing stripe rust resistance. QTL mapping with Single marker analysis, Harley-Knott regression was initially carried out using R/qtl [24] and confirmation of QTL location was performed with the ICI-mapping software [25]. Haley-Knott method is based on multiple regression gives similar estimates as of maximum likelihood method but it is relatively simple and computational rapid method which makes it easier to fit models for two or more linked and/or interacting QTL. Both R/qtl and ICIM statistical softwares are widely used as they are freely available and computationally faster. Candidate genes in the QTL interval were identified using wheat genome RefSeq v1.0 annotation (IWGSC 2018).

## Supporting information

S1 Fig Schematic representation of plant material development. (TIF)

S1 File (PDF)
