# Supplementary figures and images for: Identification of a novel stripe rust resistance gene from the European winter wheat cultivar ‘Acienda’: A step towards rust proofing wheat cultivation
Source: PLoS One. 2022 Feb 16;17(2):e0264027. doi: 10.1371/journal.pone.0264027 (PMC8849526; doi:10.1371/journal.pone.0264027)

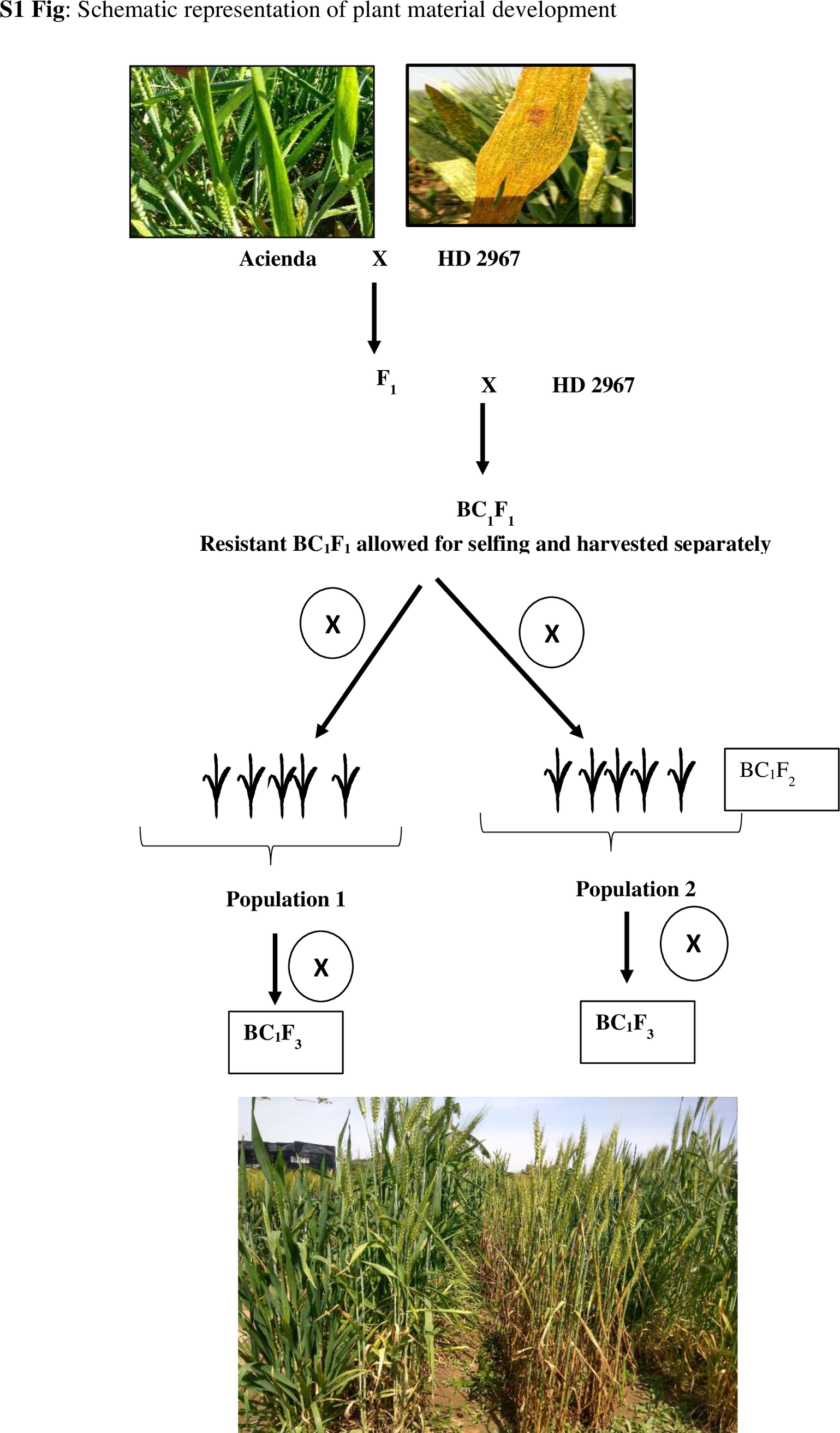

Supplement: S1 Fig — (TIF) [file pone.0264027.s001.tif]
