# Supplementary material for: Identification of a novel stripe rust resistance gene from the European winter wheat cultivar ‘Acienda’: A step towards rust proofing wheat cultivation
Source: PLoS One. 2022 Feb 16;17(2):e0264027. doi: 10.1371/journal.pone.0264027 (PMC8849526; doi:10.1371/journal.pone.0264027)
Supplement: S1 File — (PDF) [file pone.0264027.s002.pdf]

S1 Table: Number of polymorphic markers between Acendia and HD2967 wheat genotypes and their chromosomal position

Chromosome	Number of polymorphic markers	Chromosome	Number of polymorphic markers
1A	106	4D	5
1B	121	5A	161
1D	38	5B	103
2A	121	5D	10
2B	195	6A	144
2D	92	6B	96
3A	115	6D	14
3B	230	7A	145
3D	7	7B	58
4A	95	7D	32
4B	80	Unmapped	391

S2 Table: List of candidate NBS-LRR/pathogen resistance genes along with their putative function and gene length from the detected chromosome 1A physical interval based on annotations available in RefSeq v1.s

Sr. No.	Genes	Putative function	Gene length (bp)	Start (bp)	End (bp)
1	TraesCS1A01G008100.1	Powdery mildew resistance protein	4441	4497769	4502210
2	TraesCS1A01G008300.1	Pm3-like disease resistance protein	4707	4549923	4554630
3	TraesCS1A01G009300.1	Pm3-like disease resistance protein	3776	5502205	5505981
4	TraesCS1A01G009300.2	Pm3-like disease resistance protein	3776	5502205	5505981
5	TraesCS1A01G009400.1	Pm3-like disease resistance protein	4433	5645613	5650046
6	TraesCS1A01G009600.1	Pm3-like disease resistance protein	3607	6008284	6011891
7	TraesCS1A01G009900.1	Disease resistance protein (NBS-LRR class) family	5625	6152712	6158337
8	TraesCS1A01G009900.2	Disease resistance protein (NBS-LRR class) family	5625	6152712	6158337
9	TraesCS1A01G010100.1	Pm3-like disease resistance protein	682	6294153	6294835
10	TraesCS1A01G010400.1	Pm3-like disease resistance protein	4708	6336596	6341304
11	TraesCS1A01G010400.2	Pm3-like disease resistance protein	4708	6336596	6341304
12	TraesCS1A01G007200LC.1	Disease resistance protein RPP13	620	3776618	3777238
13	TraesCS1A01G007300LC.1	Disease resistance protein RPP13	1367	3845783	3847150
14	TraesCS1A01G011400LC.1	Disease resistance protein	584	4215558	4216142
15	TraesCS1A01G011900LC.1	Disease resistance family protein / LRR family protein	2953	4661007	4663960
16	TraesCS1A01G013300LC.1	Powdery mildew resistance protein Pm3	710	5523259	5523969
17	TraesCS1A01G013400LC.1	Pm3-like disease resistance protein	1112	5525956	5527068
18	TraesCS1A01G014400LC.1	Powdery mildew resistance protein Pm3	614	6011906	6012520
